# Multicenter Intestinal Current Measurements in Rectal Biopsies from CF and Non-CF Subjects to Monitor CFTR Function

**DOI:** 10.1371/journal.pone.0073905

**Published:** 2013-09-10

**Authors:** John P. Clancy, Rhonda D. Szczesniak, Melissa A. Ashlock, Sarah E. Ernst, Lijuan Fan, Douglas B. Hornick, Philip H. Karp, Umer Khan, James Lymp, Alicia J. Ostmann, Amir Rezayat, Timothy D. Starner, Shajan P. Sugandha, Hongtao Sun, Nancy Quinney, Scott H. Donaldson, Steven M. Rowe, Sherif E. Gabriel

**Affiliations:** 1 Department of Pediatrics, Cincinnati Children’s Hospital Medical Center and the University of Cincinnati, Cincinnati, Ohio, United States of America; 2 Cystic Fibrosis Foundation, Bethesda, Maryland, United States of America; 3 Department of Internal Medicine, University of Iowa, Iowa City, Iowa, United States of America; 4 Department of Medicine, University of Alabama at Birmingham, Birmingham, Alabama, United States of America; 5 Department of Biostatistics, Seattle Children’s Hospital, Seattle, Washington, United States of America; 6 Genentech, Inc., San Francisco, California, United States of America; 7 Department of Pediatrics, University of Iowa, Iowa City, Iowa, United States of America; 8 Department of Medicine, University of Alabama at Birmingham, Birmingham, Alabama, United States of America; 9 Department of Medicine, University of North Carolina, Chapel Hill, North Carolina, United States of America; 10 N30 Pharmaceuticals, Aurora, Colorado, United States of America; University of Pittsburgh, School of Medicine, United States of America

## Abstract

Intestinal current measurements (ICM) from rectal biopsies are a sensitive means to detect cystic fibrosis transmembrane conductance regulator (CFTR) function, but have not been optimized for multicenter use. We piloted multicenter standard operating procedures (SOPs) to detect CFTR activity by ICM and examined key questions for use in clinical trials. SOPs for ICM using human rectal biopsies were developed across three centers and used to characterize ion transport from non-CF and CF subjects (two severe CFTR mutations). All data were centrally evaluated by a blinded interpreter. SOPs were then used across four centers to examine the effect of cold storage on CFTR currents and compare CFTR currents in biopsies from one subject studied simultaneously either at two sites (24 hours post-biopsy) or when biopsies were obtained by either forceps or suction. Rectal biopsies from 44 non-CF and 17 CF subjects were analyzed. Mean differences (µA/cm^2^; 95% confidence intervals) between CF and non-CF were forskolin/IBMX=102.6(128.0 to 81.1), carbachol=96.3(118.7 to 73.9), forskolin/IBMX+carbachol=200.9(243.1 to 158.6), and bumetanide=-44.6 (-33.7 to -55.6) (*P*<0.005, CF vs non-CF for all parameters). Receiver Operating Characteristic curves indicated that each parameter discriminated CF from non-CF subjects (area under the curve of 0.94-0.98). CFTR dependent currents following 18-24 hours of cold storage for forskolin/IBMX, carbachol, and forskolin/IBMX+carbachol stimulation (n=17 non-CF subjects) were 44%, 47.5%, and 47.3%, respectively of those in fresh biopsies. CFTR-dependent currents from biopsies studied after cold storage at two sites simultaneously demonstrated moderate correlation (n=14 non-CF subjects, Pearson correlation coefficients 0.389, 0.484, and 0.533). Similar CFTR dependent currents were detected from fresh biopsies obtained by either forceps or suction (within-subject comparisons, n=22 biopsies from three non-CF subjects). Multicenter ICM is a feasible CFTR outcome measure that discriminates CF from non-CF ion transport, offers unique advantages over other CFTR bioassays, and warrants further development as a potential CFTR biomarker.

## Introduction

Cystic fibrosis (CF) is the most common lethal genetic disease in the Caucasian population, with autosomal recessive inheritance and an incidence of 1:3000 US births [1]. Cystic fibrosis is caused by mutations in the cystic fibrosis transmembrane conductance regulator (*CFTR*) gene, which encodes the CFTR ATP-binding cassette protein. CFTR is a chloride and bicarbonate anion channel and regulates the transport of salt and water across several epithelia [2–7]. The *CFTR* gene has >1800 disease-causing mutations, with deletion of phenylalanine at position 508 found in >85% of CF patients. F508del-CFTR is rapidly degraded in the 26S proteosome, with little if any mature protein reaching the plasma membrane [8–11]. The F508del-CFTR mutation is an appropriate target for drug development as recent results indicate that small molecules that modulate F508del-CFTR maturation (‘correctors’) demonstrate modest bioactivity in Phase II clinical trials [12].

There are two biomarkers of CFTR function that have been commonly used to detect the activity of CFTR modulators: sweat chloride (Cl^-^) and nasal potential difference (NPD) [13] [12,14–20]. These bioassays have demonstrated variable sensitivity to detect CFTR modulator bioactivity, and both biomarkers have advantages and shortcomings. For example, while sweat Cl^-^ is feasibly performed in subjects from infancy through adulthood, it is an indirect measure of CFTR function that is inextricably linked to sodium absorption through the epithelial sodium channel (ENaC) [13]. Sweat Cl^-^ values can be influenced by a number of external factors such as age, salt intake, aldosterone and other hormone levels, skin integrity, and additional medical conditions (e.g., ectodermal dysplasia, thyroid function) that are independent of CFTR activity. NPD is the only CFTR biomarker commonly used in clinical trials that fully isolates CFTR activity, thereby providing a high degree of specificity for functional CFTR at the plasma membrane [20–22]. However, the test is technically difficult to perform, may be insensitive to small changes in CFTR function, and is less feasible in children who cannot cooperate with the test. Thus, additional CFTR biomarkers that are sensitive and isolate CFTR activity, are highly discriminatory between CF and non-CF individuals, can be easily performed in young CF patients, and monitor CFTR in a target organ with disease manifestations are needed.

One assay that may address many of these limitations is Intestinal Current Measurements (ICM) from rectal biopsy samples [23–30]. This method is attractive since similar measures of ion transport (e.g., short-circuit current, I_sc_) with commercially available equipment are commonly used to quantify CFTR activity in preclinical model systems (e.g., CF and non-CF mice and pigs) [31–36]. Furthermore, ex vivo ICM is a direct assay to isolate and quantify CFTR dependent currents compared with in vivo measures of nasal potential difference or Na^+^ and Cl^-^ ion concentrations (that are the product of CFTR activity). CFTR is expressed at high levels in the rectum, the tissue is a target organ of disease that is not altered by CF manifestations or progression, and the biopsies are studied ex vivo, all of which provide flexibility in the reagents that can be used to detect and quantify CFTR activity.

ICM studies of human rectal tissue have been examined for nearly two decades, predominately in European and more recently South American CF care and research sites [27,29,37] [38]. This experience indicates that the assay is safe and well tolerated, that ICM is sensitive and specific for CFTR function, and it is highly discriminatory between severe CF (with no detectable CFTR activity), mild CF (with residual CFTR activity), and non-CF (normal CFTR function) [25,30] [23,38]. Rectal biopsies can be performed safely in infants and are routinely used to diagnose conditions in the neonatal period such as Hirschsprung’s disease. Together, these data provide strong rationale to examine the feasibility of this assay in a multicenter format prior to clinical studies of CFTR modulators.

To date there have been no studies of ICM that have used common SOPs across study centers. This lack of standardized practices could impede its use as a biomarker in future clinical trials, where standardized CFTR biomarker procedures (sweat Cl^-^, NPD) are important contributors to the successful study of CFTR modulators [18,20]. It is also unknown whether the electrophysiologic aspects of the assay (which require specialized equipment and training) can be centralized (i.e., can biopsies performed at a one site be studied by ICM at a second site) and whether different biopsy methods (e.g., use of suction or forceps) produce similar ICM data. In the current study, common SOPs for ICM performance were used across three CF research sites to generate data regarding ICM performance in CF patients with severe mutations compared with non-CF controls. The SOPs were then used to examine questions relevant to next steps in biomarker development, including the effects of cold storage on ICM results, the feasibility of performing ICM on biopsied tissue at a second site (relative to the site of origin), and ICM characteristics of rectal biopsies obtained by suction and forceps techniques.

## Methods

### Ethics statement

This study was approved by the local institutional review board at each study site, and all clinical investigation was conducted according to the principles expressed in the Declaration of Helsinki. All study subjects signed informed consents for study participation.

### Study subjects

Initial studies were completed in non-CF subjects to demonstrate assay proficiency (five sequential subjects) using a central data interpreter. Subsequently, CF and non-CF subjects were enrolled prospectively using the common SOPs developed by the study team. Non-CF subjects undergoing scheduled lower endoscopy for clinical purposes (e.g., cancer screening) were targeted for enrollment, and bowel preparation was per the practices of the treating gastroenterologist. All CF patients underwent the biopsy for research purposes, and performed a self-administered enema (in each 118 mL delivered dose, monobasic sodium phosphate monohydrate = 19 gm, and dibasic sodium phosphate heptahydrate = 7 gm) prior to the procedure. CF patients were offered sedation, and a simple questionnaire assessing their tolerance of the procedure was administered following completion.

### SOP development

Common equipment, biopsy methods, tissue dissection, voltage clamp, and reagents were defined based on review of prior publications, discussions with personnel experienced in ICM performance, and testing of conditions [23,25,27,29,30]. Personnel from each of the study sites underwent common onsite training in tissue dissection, mounting, and Ussing chamber assessment.

### Biopsy

Forceps biopsies were performed using the Olympus endojaw pinless beveled biopsy forceps (FB-230U, Olympus medical systems, Japan) under direct visualization by a trained gastroenterologist. Suction biopsies were performed using the C-Rbi2 rectal biopsy system (Aus systems Pty Ltd, South Australia, Australia). Biopsies were placed in iced RPMI 1640 buffer, and transported directly to the ICM technician for dissection and mounting. The time between biopsy and mounting was typically less than one hour.

### Equipment and reagents

Identical voltage clamps and tissue mounts were used at all study sites (Physiologic Instruments, San Diego, CA), including VCC MC8 clamps and chambers. Tissues were warmed to 37^°^C by a circulating pump and continuously gassed with 95% O2: 5% CO2 in RPMI 1640 media + 25 mM HCO_3_ unless indicated otherwise, when Ringers buffer (NaCl 120 mM, NaHCO_3_ 25 mM, KH_2_PO_4_ 3.33 mM, K_2_HPO_4_ 0.83 mM, CaCl_2_.2H_2_O 1.2 mM, MgCl_2_.6H_2_O 1.2 mM, and gluconic acid 10 mM) replaced RPMI media. Tissue was dissected under a dissection microscope, removing the muscularis mucosa followed by mounting in P2302T, 0.33cm^2^ sliders. The tissue was then placed in the Ussing chambers, and voltage clamped to monitor I_sc_ as previously described [27]. Tissue was treated with 10 µM indomethacin (mucosal and serosal compartments) to reduce CFTR dependent Cl^-^ secretion to baseline. Following stabilization of currents (~30 min) the tissue was treated with amiloride (100 µM, mucosal compartment) to block Na^+^ absorption for 15 min. Tissues were then stimulated with 10 µM forskolin + 100 µM IBMX (mucosal and serosal compartments) to raise intracellular cAMP and monitored for 20 min. Tissues were then stimulated with carbachol (100 µM, serosal) to activate basolateral K^+^ channels and augment CFTR-dependent currents. These maneuvers have been shown to isolate CFTR activity, producing a large CFTR-dependent Cl^-^ secretory current (serosal to mucosal direction) that is evident in the presence of functional CFTR at the mucosal plasma membrane [23,25,27]. In the absence of CFTR, these maneuvers can produce a small secretory K^+^ current with a downward deflection in the I_sc_. Following current stabilization (15-20 min), bumetanide (100 µM) was added to the serosal compartment to block the Na^+^/K^+^/2Cl^-^ co-transporter. This inhibited CFTR-dependent Cl^-^ transport in the presence of CFTR (downward deflection in I_sc_), or produced an upward deflection in the absence of CFTR (inhibition of K^+^ secretion). Examples of tracings from non-CF and CF subjects are included in Figure S1 in File S1. The CFTR blockers CFTR_inh-172_ (10-50 µM) and GLYH101 (50-100 µM) failed to predictably inhibit CFTR currents in human rectal biopsy tissue (Figure S2 in File S1), and thus were not routinely used in our studies.

Forskolin, 3-isobutyl-1-methylxanthine (IBMX, a nonspecific phosphodiesterase inhibitor), carbachol, and bumetanide were obtained from Sigma-Aldrich, St. Louis, MO, aliquoted into single use tubes, and stored frozen until the biopsies were studied. RPMI 1640 media was obtained from Invitrogen, Carlsbad, CA. CFTR_inh172_ and GLYH101 were purchased through the CFFT Compound Distribution Program.

### Data collection

Prior to analyses, all data were interpreted by a blinded reader (site and diagnosis). Each biopsy sample had up to five I_sc_ parameters calculated, including the change in I_sc_ following amiloride, forskolin/IBMX, carbachol, forskolin/IBMX + carbachol, and bumetanide additions. Mean values were calculated in subjects with multiple biopsies obtained at the same study site or on the same day.

### Statistical analysis

A descriptive analysis was performed with calculation of means, medians, and standard deviations for continuous data and percentages for categorical data. Analyses of SOP effectiveness, including the impact of storage conditions, study site agreement and extraction method agreement, were conducted using I_sc_ parameters from biopsies of only non-CF subjects. The impact of storage condition (cold versus fresh tissue) on each I_sc_ parameter was examined using the paired t-test and intraclass correlation coefficient (ICC) analysis [39], where fresh tissue was considered to be the gold standard. In addition to the paired t-test and ICC, measurement agreement between sites with respect to each I_sc_ parameter was assessed using Bland-Altman analysis [40]; the site of biopsy origin was treated as the gold standard. Due to the limited number of subjects providing forceps and suction biopsies, descriptive statistics for the two methods are shown. For comparisons of tissue resistance in forceps vs suction biopsies, paired t-tests were used. Data transformations were used for I_sc_ parameters to achieve normality assumptions for inferential analyses.

Differences between CF and non-CF subjects with respect to continuous measures were assessed using the Wilcoxon Mann-Whitney test. The diagnostic performance of each I_sc_ parameter at distinguishing CF and non-CF tissues was examined using Receiver Operator Characteristic (ROC) curves; results are reported as Area Under the Curve (AUC). Power analyses were used to determine the capability of ICM and NPD parameters to detect statistically significant differences in increasing levels of CFTR function. The approach was based on a one-sided paired t-test for mean differences observed pre- and post-observation with alpha = 0.05, assuming intrasubject correlation of 0.50 between observation periods. The intersubject SD observed from the study data was used as the upper bound on calculations.

To assess variance in CFTR responses (intra-site and inter-site), Analysis of variance (ANOVA) was performed. This produced the model sum-of-squares (inter-site variation) and error sum-of-squares (intra-site variation), along with the total sum-of-squares. This information was then converted to variances (SD^2^ / [n -1]).

Agreement analyses for I_sc_ parameters have been back-transformed to original scale. Model adequacy was verified with each agreement assessment using residual diagnostics. All results are reported as mean (95% CI) for continuous data and N (%) for categorical data unless stated otherwise. Analyses were performed using R version 2.13 (R Foundation for Statistical Computing, Vienna, Austria) and SAS version 9.3 (SAS Institute, Cary, NC).

## Results

### Development of SOPs for ICM performance

The studies supporting SOP development are summarized in the online Supporting Information, including examples of CF and non-CF tracings (Figure S1 in File S1), the effects of CFTR blockers on CFTR currents (Figure S2 in File S1), the impact of indomethacin on ICM parameters (Figure S3 in File S1), comparison of buffer conditions (Figure S4 in File S1), and stimulation with CFTR-activating conditions (Figure S5 in File S1). Guidelines for SOP development were based on conversations with experts in the field and published reports coupled with an overriding goal of a simple, straightforward assay that utilized common equipment and software [23,25,27,29,30]. All studies supporting SOP development were performed in non-CF subjects (n = 5-6 for each condition). Based upon these results, the reagents and conditions shown in Figure S1 in File S1 were used for the ensuing studies.

### Prospective multi-center ICM in CF and non-CF subjects

A total of 45 non-CF and 12 F508del/F508del subjects were enrolled in this component of the study, and data was available from 44 non-CF subjects (%) and 11 CF subjects (%). Consent from one CF and one non-CF subject was withdrawn prior to biopsy (non-CF subject due to discovery of ulcerative colitis; CF subject due to painful hemorrhoids). In addition, six CF subjects were enrolled in a parallel protocol at one site (genotypes were F508del/F508del for five subjects, E60X/621+1G-T for the sixth subject). All study conditions were identical in this parallel study except for the use of Ringers buffer rather than RPMI 1640 buffer. Based on the similar ICM results comparing Ringer’s and RPMI buffer (Figure S4 in File S1), data from these additional CF subjects was included in the full analysis.

Subject demographics are shown in Table 1. As expected based on the enrollment populations, the non-CF subjects were 25 years older than the CF subjects, and had a more diverse racial and ethnic background. Sixty-five percent of CF subjects were male whereas forty-five percent of non-CF subjects were male. Enrollment was similar across the three study sites, ranging between 25%-44% of the total enrollment. A total of 291 biopsies were obtained from all study subjects, with 227 (78%) providing interpretable results based on central interpretation criteria (stable baseline current and tissue resistance). A total of six biopsies were inverted (2%) based on directional responses to agonists and post-analysis un-blinding to diagnosis (CF or non-CF). Of the 44 non-CF subjects, 37 had bowel preparation with polyethylene glycol (PEG), with a minority having pre-procedure preparation with magnesium citrate with or without PEG (n = 4) or not specified (n = 3). A total of five AEs were reported, including post-procedure bleeding (n = 4, all self-resolved) and pain from the procedure (n = 1, self-resolved). All AEs were assessed as probably related to the procedure by the gastroenterologist. The median number of biopsies per subject was four (range 2-8), and all interpretable biopsies were averaged to provide one value per subject for each of the ICM parameters.

**Table 1:** Demographics characteristics by study group.

	non CF	CF	All
	n = 44	n = 17	n = 61
Age - yrs			
Mean (SD)	56.0 (9.97)	31.0 (8.59)	49.1 (14.8)
Median	54.4	28.2	51.3
Min, Max	26.0, 76.9	21.5, 50.1	21.5, 76.9
Age distribution – no. (%)			
18 - ≤ 24	0	3 (18)	3 (5)
24 - ≤ 36	2 (5)	9 (53)	11 (18)
36 - ≤ 48	4 (9)	4 (24)	8 (13)
> 48	38 (86)	1 (6)	39 (64)
Sex – no. (%)			
Male	20 (45)	11 (65)	31 (51)
Female	24 (55)	6 (35)	30 (49)
Race – no. (%)			
no. with race recorded	44	17	55
Caucasian	35 (80)	16 (94)	45 (82)
African American	8 (18)	1 (6)	9 (16)
Other	1 (2)	0	1 (2)

The ICM results for the CF and non-CF subjects across all study sites are summarized in Table 2. Tissue resistance was similar for both the CF and non-CF groups (*P* = NS). Statistically significant differences in I_sc_ responses between the CF and non-CF subjects were observed following amiloride (*P* = 0.002), forskolin/IBMX (*P* < 0.001), carbachol (*P* < 0.001), forskolin/IBMX + carbachol (*P* < 0.001), and bumetanide (*P* < 0.001). The absolute change in amiloride-sensitive current was greater in the non-CF subjects compared with the CF subjects, while the changes in all other ICM parameters were as predicted for the absence of CFTR-function in the CF compared with the non-CF sample.

**Table 2:** Summary of changes in Na^+^ and Cl^-^ ICM currents (µA/cm^**2**^).

	**CF**	**Non-CF**	**Difference**
**Condition**	**n = 17**	**n = 44**	**(CF vs non-CF)**
Change Amiloride (µA/cm^2^)			
Mean (SD)	-10.5 (29.08)	-21.4 (27.79)	Mean diff = -11.0
Median	-1.1	-12.3	95% CI = (6.4, -28.4)
Min, Max	-112.5, 7.9	-159, 6	*P* = 0.002*
Change cAMP (forskolin/IBMX)** (µA/cm^2^)			
Mean (SD)	-5.0 (12.67)	99.5 (71.84)	Mean diff = 102.6
Median	-0.9	92	95% CI = (128.0, 81.1)
Min, Max	-35.7, 16.4	6.1, 269.4	*P* < 0.001*
Change CCh (µA/cm^2^)			
Mean (SD)	5.9 (10.21)	102.2 (69.21)	Mean diff = 96.3
Median	3.9	97.26	95% CI = (118.7, 73.9)
Min, Max	-12.7, 28.1	0.8, 318.7	*P* < 0.001*
Change cAMP + CCh (µA/cm^2^)			
Mean (SD)	0.8 (14.66)	201.7 (131.98)	Mean diff = 200.9
Median	-1.8	193.1	95% CI = (243.1, 158.6)
Min, Max	-30.3, 31.9	19.5, 588.1	*P* < 0.001*
Change Bumetanide (µA/cm^2^)			
Mean (SD)	8.8 (9.7)	-35.8 (31.35)	Mean diff = -44.6
Median	6.4	-27.4	95% CI = (-33.7, -55.6)
Min, Max	-2.6, 30.9	-166.2, 3.3	*P* < 0.001*

cAMP, forskolin/ IBMX; CCh, carbochol; CI, confidence interval

* *P* values were obtained using the Mann-Whitney test

** Represents the primary clinical endpoint: difference in mean change in current for cAMP-stimulated tissue between CF and non-CF participants.

ICM data from 16 CF and 41 non-CF subjects were available for analysis.

**Figure 1:**
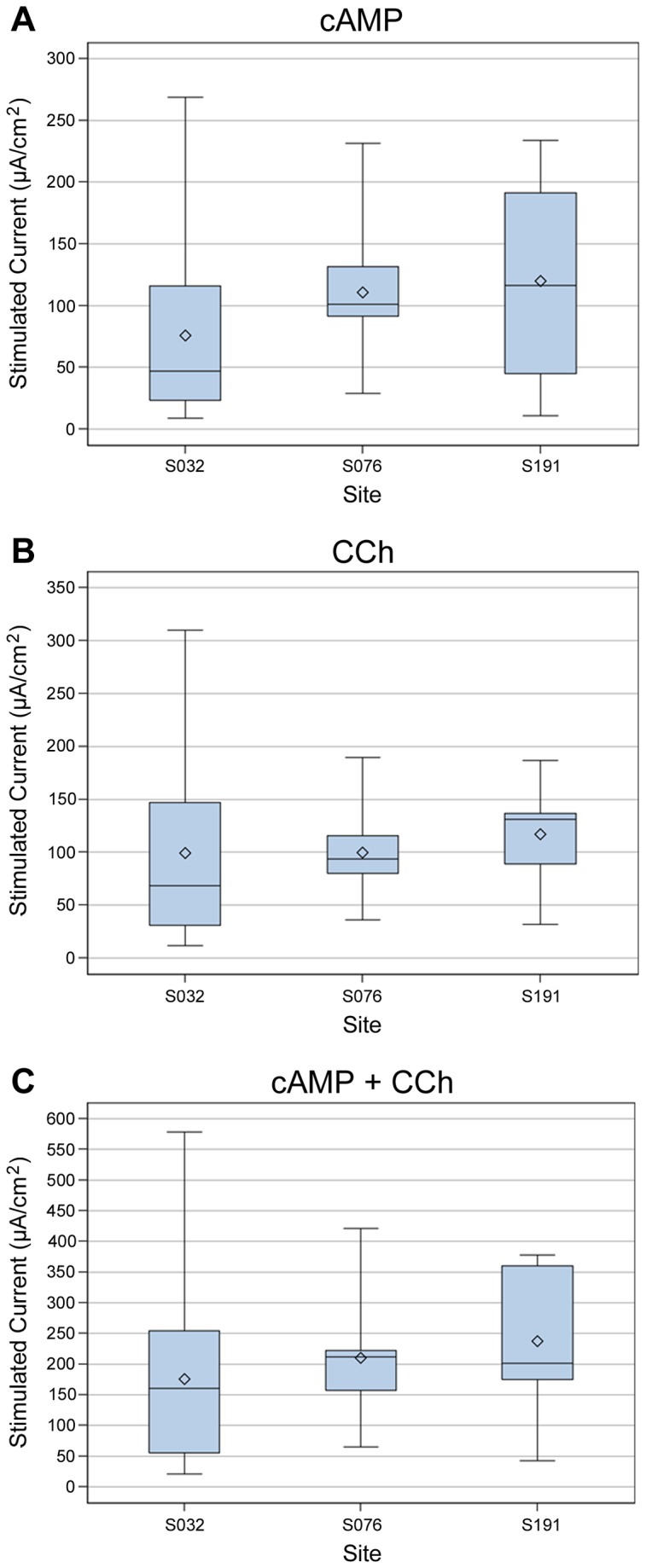
Individual ICM responses across study sites. Boxplots of CFTR responses in non-CF subjects segregated by site (site identifiers S032, S076, S191). Whiskers are minimum and maximum values, boxes included data within 25^th^-75^th^ percentiles, the horizontal line is the median, and the diamond is the mean. **A**. Change in I_sc_ following cAMP stimulation (10 µM forskolin/100 µM IBMX). **B**. Change in I_sc_ following carbachol (CCh) stimulation (100 µM, basolateral). **C**. Change in I_sc_ following cAMP + CCh stimulation (10 µM forskolin, 100 µM IBMX, 100 µM CCh).

The CFTR-dependent responses for non-CF subjects at each site (forskolin/IBMX, carbachol, forskolin/IBMX + carbachol) and their variances (within and across sites) are shown in Figure 1A–C and Table 3. Mean and median values for the three conditions were similar across the three study sites for the carbachol and forskolin/IBMX + carbachol-stimulated currents, and one site (S032) in general demonstrated higher variability for all three stimuli compared with the other two sites (S076 and S191). Table 3 summarizes the contribution of inter-site (between) and intra-site (within) variability to total variability for the same parameters shown in Figure 1. Greater than 90% of total variance for all three CFTR measures was due to intra-site variability, and not due to site-specific differences in assay conduct or results.

**Table 3:** Camparison of intersite and intrasite variability of CFTR-dependent currents in non-CF subjects using variances.

**Variable**	**Inter-Site Variability (% of Total)**	**Intra-Site Variability (% of Total)**	**Total Variability (% of Total)**
cAMP	384 (8.6)	4069 (91.4)	4453 (100)
CCh	64 (1.6)	3945 (98.4)	4009 (100)
cAMP + CCh	648 (4.5)	13879 (95.5)	14527 (100)

cAMP, forskolin/ IBMX; CCh, carbochol

**Figure 2:**
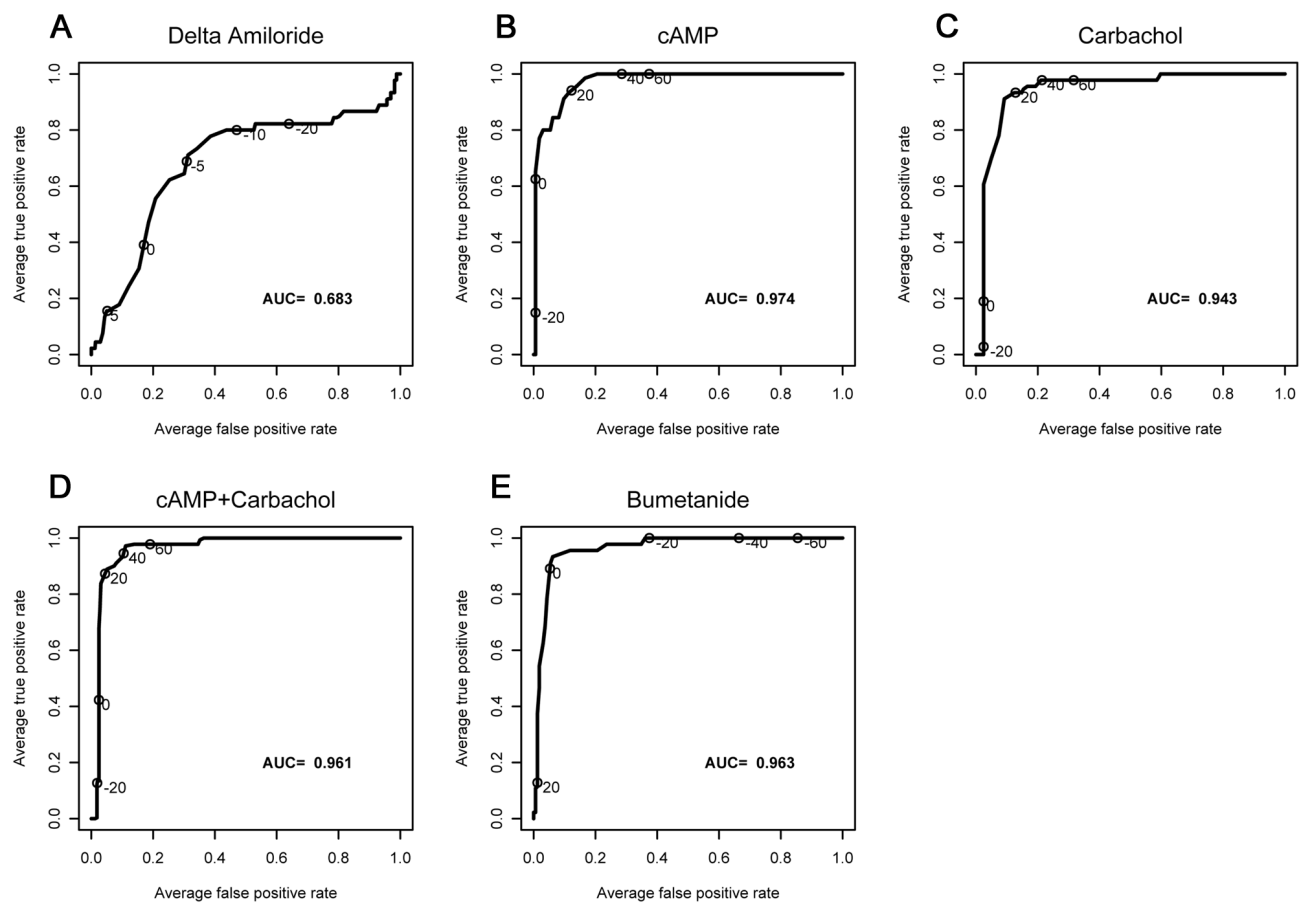
ROC curve analysis. Note that CF participants are coded as one, and non-CF participants are coded as zero. Average true-positive rate is the sensitivity of the current to detect CF participants, whereas the average-false positive rate (one-specificity) marks the cutoff whereby non-CF participants are falsely determined as CF. AUC values varied from 0.946-0.978 for the three CFTR-specific measurements (forskolin/IBMX (cAMP), carbachol (CCh), cAMP + CCh).

**Figure 3:**
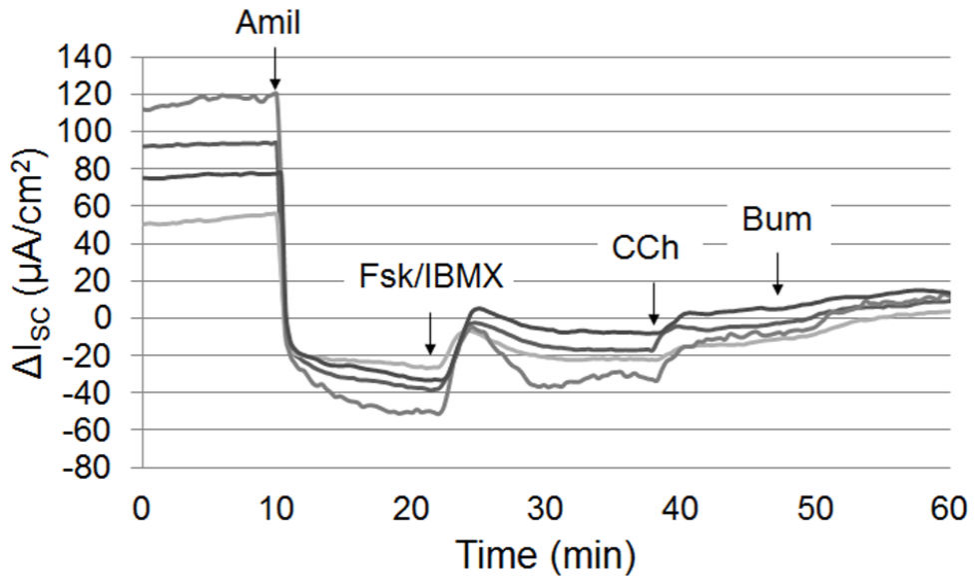
Partial CFTR function detected in F508del/F508del CF subject. Representative tracing from an F508del/F508del subject with functional F508del CFTR. Adding forskolin/IBMX to raise cAMP increased the current > 30µA/cm^2^ which was further augmented by carbachol.

ROC curves are shown in Figure 2. All ICM parameters measured except the response to amiloride produced similar ROC curves, with AUC values ranging from 94–98%, indicating that the parameters differentiated CF subjects from non-CF subjects well. Of the parameters examined, the responses to forskolin/IBMX, carbachol, and forskolin/IBMX+carbachol had similar sensitivity. ICM was also capable of detecting partial CFTR activity. Figure 3 depicts the ICM tracing from one F508del/F508del subject (male, age 37 years, sweat Cl^-^ = 113 mMol, pancreatic insufficient, FEV_1_ = 63%) showing features of functional CFTR, including a clear Cl^-^ upward deflection following stimulation with forskolin/IBMX (compare with non-CF result in Figure S1 in File S1). The total change in I_sc_ produced by forskolin/IBMX+carbachol was 15.8% of the mean wtCFTR response (non-CF subjects).

Based on the results of these pilot studies, we examined the power of ICM to detect increasing levels of CFTR function based on monitoring the ICM response to forskolin/IBMX, carbachol and forskolin/IBMX + carbachol. We compared this with the capacity of NPD to detect similar increases in CFTR activity (change in PD following perfusion with zero Cl^-^ + 10 µM isoproterenol, a well-defined measure of CFTR activity used to successfully detect CFTR modulator activity in CF patients [14,15,18,20,41]. The NPD data was derived from qualification tracings submitted by 29 NPD study sites that were centrally interpreted at the Center for CFTR Detection at the University of Alabama at Birmingham (n = 144 non-CF tracings and 135 CF tracings) [12,18,20,42]. All subjects had a diagnosis of CF based on standard criteria (sweat Cl^-^ > 60 mMol, organ manifestations of CF) and received CF care at the submitting study sites. Genotype information was not included with the NPD tracings. The results of these comparisons are summarized in Table 4. All three ICM parameters demonstrated substantially greater power than the NPD to detect low levels of CFTR function.

**Table 4:** Comparison of NPD and ICM power to detect increasing levels of CFTR function.

CFTR Measurement	Mean Non-CF response	Mean CF response	Difference (SD)	Percent of non-CF response	Projected Treatment Effect	Estimated Effect Size	Number of Subjects	
							80% Power	90% Power
**NPD:**								
Zero Cl/Iso (mV)	-22.41	1.18	23.49 (3.94)	10	2.35	0.59	19	26
				20	4.70	1.19	6	8
				30	7.05	1.79	4	5
				40	9.40	2.39	3	4
				50	11.75	2.98	3	3
**ICM:**								
cAMP (µA/cm^2^)	-99.50	-5.00	94.50 (12.67)	10	9.45	0.75	13	17
				20	18.90	1.49	5	6
				30	28.35	2.24	4	4
				40	37.80	2.98	3	3
				50	47.25	3.73	3	3
CCh (µA/cm^2^)	-102.20	5.90	108.10 (10.21)	10	10.81	1.06	8	10
				20	21.62	2.11	4	4
				30	32.43	3.18	3	3
				40	43.24	4.24	3	3
				50	54.05	5.29	3	3
				60	64.86	6.52	2	3
cAMP + CCh (µA/cm^2^)	-201.70	0.80	202.50 (14.66)	10	20.25	1.38	5	7
				20	40.50	2.76	3	4
				30	60.75	4.14	3	3
				40	81.00	5.53	3	3
				50	101.25	6.91	2	3

Iso, isoproterenol; CCh, carbochol

CF patient tolerance of the rectal biopsy procedure was assessed during the study using a simple questionnaire. A total of eight CF subjects completed this questionnaire post-procedure. Seven of eight subjects chose to undergo the procedure without sedation. Subjects were asked to rate the different aspects of the procedure as ‘not unpleasant’, ‘sort of unpleasant’, or ‘very unpleasant’, including preparation (enema), sigmoidoscopy, biopsy, monitoring, and patient willingness to undergo a rectal biopsy as part of a future study. The majority of patients rated the enema and sigmoidoscopy as ‘sort of unpleasant’ (62% and 71%, respectively), while the majority rated the biopsy and monitoring as ‘not unpleasant’ (71% and 100%, respectively). None of the subjects rated any of the aspects of the procedure as ‘very unpleasant’, and all subjects responded that they were willing to undergo rectal biopsy as part of future CF studies.

### Effects of cold tissue storage on ICM parameters

Since ICM performance requires significant onsite training and equipment, we examined whether the electrophysiologic aspects of ICM could be centralized. In preparation for these studies, we examined the effects of different storage conditions on tissue viability and CFTR activity in non-CF subjects. First, four separate media conditions for cold storage were compared (summarized in Figure S6 in File S1). The results indicated that biopsies stored at 4^°^C for 18 hours in RPMI 1640 media retained consistent CFTR dependent responses to agonists. Subsequently, 17 non-CF subjects underwent forceps-based biopsy, with half of the biopsies studied immediately and the other half studied after onsite storage at 4^°^C (RPMI media + antibiotics) for 18-24 hours. The results are summarized in Table 5. The responses to forskolin/IBMX (10/100 µM), carbachol (100 µM), and forskolin/IBMX + carbachol following cold storage were reduced relative to responses in fresh tissue (*P* = 0.026, 0.046, and 0.039, respectively). The responses after cold storage were 44%, 47.5%, and 47.3% of the fresh-tissue responses for the three respective stimulus conditions (forskolin/IBMX, carbachol, forskolin/IBMX + carbachol).

**Table 5:** Effects of cold storage on ICM parameters (single site).

Change in I_sc_ µA/cm2 – Least Square Means (Upper C.L., Lower C.L.)			
Tissue	*cAMP	*CCh	*cAMP + CCh
Fresh	65.29 (38.23, 102.81)	88.07 (62.34, 120.06)	155.85 (106.34, 218.75)
Stored	28.70 (15.88, 47.04)	41.91 (22.03, 71.16)	73.80 (41.02, 120.58)

cAMP, forskolin/ IBMX; CCh, carbochol

* *P* < 0.05 for comparisons between fresh and stored for the different stimuli (n = 17 non-CF subjects)

### Variance of ICM CFTR parameters measured in one subject at two study sites

**Figure 4:**
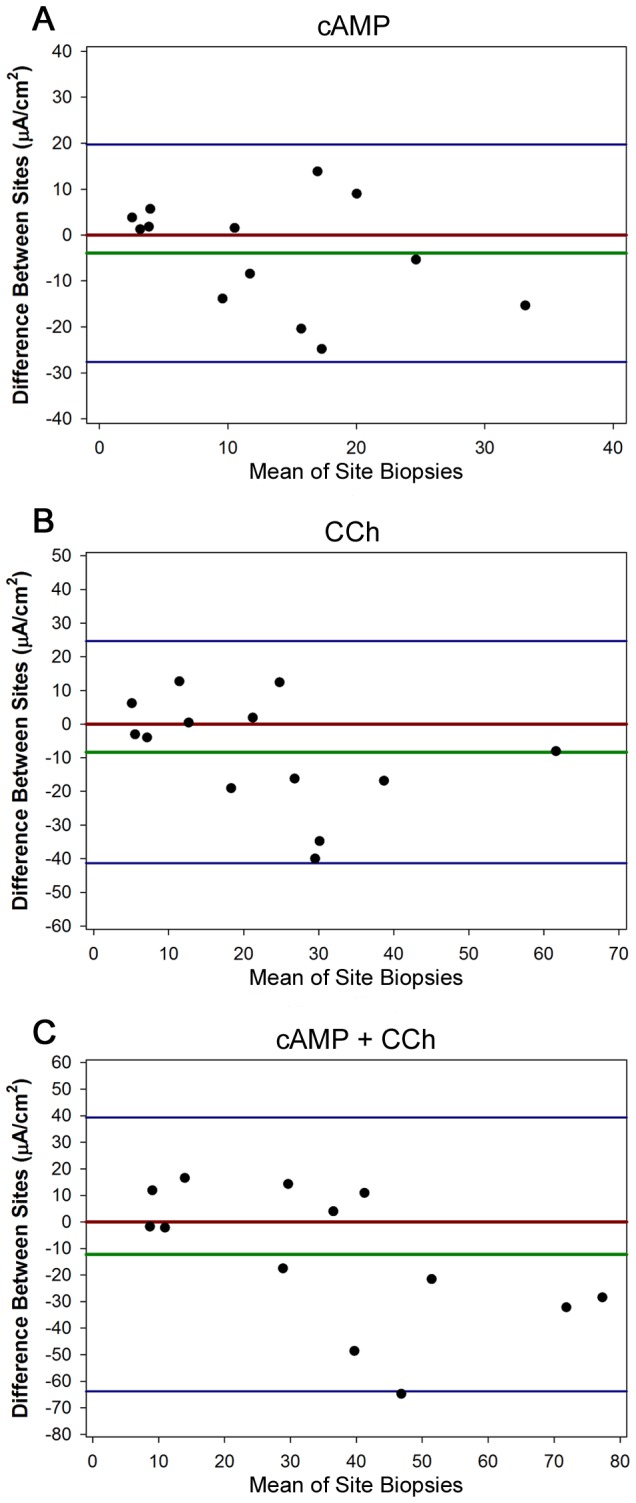
Bland-Altman plots of ICM responses for biopsies from single subjects studied at two sites simultaneously after cold storage. Difference between **A**. forskolin/IBMX (cAMP), **B**. carbachol, or **C**. forskolin/IBMX (cAMP + carbachol) responses at site of biopsy origin and test site. Each dot is the mean ICM response for one subject (both sites). The X axis is the mean of the response (both sites), and the Y axis is the difference between the means at the two sites. The red line is the hypothetical zero difference, and the green line is the actual mean difference. Blue lines are ± 2 SDs.

Based on the observation that cold stored rectal biopsy tissue retained significant CFTR-dependent currents activity (Table 5), we examined the intrasubject variance of ICM parameters, testing biopsies from one non-CF subject studied at two ICM sites simultaneously. 14 non-CF subjects underwent forceps-based biopsy, with half of biopsies stored onsite (4-8^°^C) in RPMI 1640 media and the other half sent on ice overnight in RPMI media to a second ICM study site. Biopsies were then dissected and mounted in Ussing chambers at the two sites and studied simultaneously (range of 18-24 hours post-biopsy). The ICC coefficients for the three CFTR-dependent parameters (forskolin/IBMX, carbachol, and forskolin/IBMX + carbachol stimulated currents) were 0.403, 0.5144, and 0.494, respectively between the two sites. None of the parameters had differences between sites that reached statistical significance. Bland-Altman plots are shown in Figure 4A–C, demonstrating that the three ICM parameters had similar variance between sites independent of the magnitude of stimulated I_sc_.

### Comparison of CFTR activity in forceps and suction biopsies

Three non-CF subjects underwent both forceps-based and suction-based biopsies simultaneously, with subsequent ICM performance onsite. The I_sc_ responses to forskolin/IBMX, carbachol, and forskolin/IBMX + carbachol are shown in Figure S7 in File S1, including individual biopsy results and mean results for the three subjects (n = 12-14 biopsies per condition). No significant differences in CFTR dependent currents were observed based on the nature of biopsy for the three parameters, but there was a trend towards higher resistance in forceps biopsies relative to suction biopsies [22.82 ohm·cm^2^ (±8.85) vs 13.21 ohm·cm^2^ (±6.55) following subtraction of fluid resistance, *P* = 0.056].

## Discussion

In this prospective study, we used common SOPs for multi-center ICM performance and tested their performance in CF and non-CF subjects. Our data indicated that multiple ICM parameters performed across three study sites clearly distinguished between the CF and non-CF condition. Most notably were changes in I_sc_ produced by forskolin/IBMX, carbachol, forskolin/IBMX + carbachol, and bumetanide (Table 2). All of these parameters are dependent on CFTR at the apical plasma membrane. In addition, all of these electrophysiologic parameters appeared to have similar sensitivity to discriminate between CF subjects with severe mutations and non-CF subjects (ROC characteristics, Figure 2). This redundancy provided further assurance of discrimination by CFTR function, as numerous measures showed similar behavior by ROC analysis. The one exception was amiloride-sensitive currents, which were not particularly discriminatory between CF and non-CF subjects (see Figure 2A). This is not entirely unexpected since ENaC regulation varies throughout the large intestine and is dependent in part on local protease activity [43], as well as aldosterone and volume status. Our results using voltage-clamp conditions were in good agreement with results reported using open-circuit conditions, suggesting that the method of monitoring CFTR was not a major determinant of the measured CFTR dependent currents [25]. Due to the large difference in ICM responses between ‘severe’ CF and non-CF subjects with relatively low variance (Tables 2-4), the power of the assay to detect low levels of CFTR activity compared favorably with the NPD. Most of the variance observed in ICM represented differences within sites and not between sites (Figure 1 and Table 3). Intra-site variance for CFTR-dependent measures could be due to a number of contributors, including biologic variability between subjects, differences in biopsy depth, tissue injury during dissection, mucosal visualization during the biopsy procedure, and so on. To ensure optimal assay performance, standardization of those aspects of ICM that can be standardized is highly recommended (e.g., equipment, reagents, software), as are ongoing efforts to minimize the contributions of site variables (e.g., biopsy techniques, tissue integrity, dissection). In addition, other stimuli and ion transport blockers may be considered to reduce variability of CFTR dependent responses between subjects (e.g., histamine and DIDS to isolate CFTR activity).

Previous published work has demonstrated that low levels of murine *F508del* CFTR can be monitored by ICM, including ex vivo treatment with putative F508del corrective agents or conditions [35,36,44,45]. In addition, Bedwell and colleagues have shown that transgenic mice carrying the human *G542X* mutation in CFTR that are treated with suppressors of premature termination codons (aminoglycosides, ataluren) have detectable CFTR function in intestinal tissue studied by ICM [46–49]. These results in CF animal models support the notion that monitoring rectal biopsy current is a sensitive assay to detect restored CFTR function, either in response to ex vivo treatment of tissue with CFTR modulators (i.e., as a model system to test modulator activity) or following in vivo treatment (i.e., as an outcome measure for clinical trials). Future studies confirming the activity of CFTR modulators in rectal biopsies from CF patients (treated in vivo or ex vivo) will be necessary to determine definitively whether ICM is capable of detecting restored function of human CFTR carrying disease-causing mutations in human tissue. Rectal biopsies have been used to monitor *F508del* CFTR expression in one CFTR modulator trial (VX-809), using rectal biopsies to assess the maturation of *F508del* CFTR by immunoblot [12]. Thirty three of 89 enrolled subjects voluntarily underwent this optional endpoint in the VX-809 trial (biopsies prior to and during treatment), supporting the feasibility of incorporating rectal biopsies into interventional CF clinical studies. ICM was not included in the analysis of these biopsies, but evidence suggests that electrophysiologic measures are more sensitive than biochemical assays to detect low levels of mature *F508del* CFTR [29,50].

The studies examining CFTR responses following cold storage demonstrated that this maneuver reduced currents substantially (Table 5), but still provided CFTR-dependent currents that were outside the CF range for fresh tissue (Table 2). Biopsies from single subjects studied after cold storage at two sites demonstrated moderate correlation between sites and similar variance over a wide range of detected CFTR activity (Figure 4A–C), supporting the hypothesis that centralized performance of the electrophysiologic aspects of ICM may be potentially feasible. To optimize centralized ICM performance, minimizing the time between biopsy and electrophysiologic measurements at the second site are likely critical, including time between biopsy and transport, and the nature of sample transportation (e.g., next morning delivery). Finally, the method of biopsy (suction or forceps) did not clearly influence the capacity to detect CFTR-dependent currents (Figure S7 in File S1). Although these studies are limited in number, they do suggest that sites have flexibility in the nature of rectal biopsy performance.

Previous studies have helped to define stimuli that reflect CFTR in the colon, including ENaC blockade with amiloride, stimulation with cAMP-elevating agents to activate CFTR, and stimulation of basolateral muscarinic receptors that activate basolateral K^+^ channels to hyperpolarize the cell and drive Cl^-^ exit across the colonocyte apical membrane [23–25,27,51]. Increasing transepithelial Cl^-^ flux via stimulation of basolateral K^+^ channels is a unique feature of ICM compared with other CFTR biomarkers (e.g., sweat Cl^-^ or NPD), providing an example of how access to the basolateral membrane increases the flexibility in reagent use to optimize CFTR detection.

The protocol developed in the current study was based on features previously established by leading laboratories in intestinal physiology, incorporating aspects from different practices into the described ICM SOP. Based on communications with expertise in the field, onsite experience, and the need to standardize equipment, all sites utilized common voltage clamps, Ussing chambers, tissue sliders, and software from a single source. The recirculating chambers (as opposed to continuous volume-exchange chambers) were selected based on the availability of standardized equipment and servicing. Data generated at sites could then be sent directly to the central reading site for interpretation in a blinded fashion without concerns of software incompatibilities or conversion. Our results compare favorably with two recent studies examining ICM in CF in non-CF subjects. Hirtz and colleagues demonstrated that ICM was capable of clearly segregating non-CF subjects from CF patients with either partial or non-functional CFTR mutations [25]. The cAMP-stimulated currents reported in non-CF controls and patients with non-functional CFTR mutations were similar to those reported here (monitored under open circuit conditions). In a more recent study reported by Derichs and colleagues, currents were measured in 309 biopsies from 130 subjects with pancreatic insufficient CF, pancreatic sufficient CF, an unclear CF diagnosis, and healthy controls [30]. A cutoff of -34 µA/cm^2^ was described to discriminate between a CF and non-CF diagnosis. In agreement with this report, our results defined 95% CI surrounding the CF response to forskolin/IBMX + carbachol of -30.3 to +31.9 µA/cm^2^.

Another goal of the project was to develop conditions and a reagent sequence that would maximize detection of CFTR activity in a rapid fashion. The results described in Figures S1-S5 in File S1 support SOPs that include the pretreatment of samples with indomethacin, that CFTR dependent currents were similar when using either RPMI or Ringer’s buffer, and that sequential stimulation with reagents enhanced CFTR detection. While the durability of modulator drug effects on mutant CFTR in biopsied rectal tissue are unknown, preclinical studies with *F508del* CFTR correctors indicate that the half-life of *F508del* CFTR at the airway cell membrane is several hours [52–54]. Thus, the timeline for ex vivo assessment of CFTR developed in these studies seems unlikely to limit the capacity of ICM to detect corrected *F508del* CFTR at the colonocyte cell membrane. Unfortunately, neither CFTR_inh172_ nor GLYH101 were consistently effective blockers of CFTR currents in our studies (Figure S2 in File S1). The only anion channel that has been shown to play a major role in the colonocyte apical membrane to date is CFTR ( [55]). We speculate that mucus (or other molecules) on the apical surface of the colonic epithelium may bind the lipophilic blockers or limit access to CFTR through other molecular interactions. In addition, CFTR expression in the colon is highest in the base cells of the colonic crypts, which may limit accessibility of channel blockers [56]. The Na^+^/K^+^/2Cl^-^ cotransporter blocker bumetanide was thus chosen to inhibit the stimulated currents, although its blockade also was incomplete. The direction of I_sc_ inflection following bumetanide differed between the CF and non-CF subjects, likely reflecting the nature of the dominant operative ion transport pathway that was sensitive to bumetanide (CFTR-dependent Cl^-^ secretion in non-CF subjects, K^+^ secretion in CF). This feature, coupled with the direction of inflection following amiloride (which defines the apical membrane of both CF and non-CF subjects) helped provide assurance of tissue mounting and underlying diagnosis. An example of this is shown in Figure 3, in which the data support the hypothesis that this subject has detectable *F508del* CFTR at the colonic cell membrane. The ICM shows a strong downward deflection with amiloride (confirming correct orientation), a significant upward deflection in response to forskolin/IBMX, and a mixed response to carbachol. Limitations of this study include the relatively small number of CF subjects enrolled, the lack of CF patients with pancreatic sufficiency and/or partial function mutations, and the lack of pediatric subjects. As this study was performed solely for the purpose of assay development across three sites and was not designed to provide diagnostic information to the study population, enrollment of large numbers of adult or pediatric CF patients was not thought to be ethical. In addition, insufficient numbers of adult patients with pancreatic sufficiency or partial function mutations were available to draw conclusions regarding ICM performance in this CF subpopulation. In addition, the lack of acute efficacy of CFTR blockers (CFTR_inh172_ and GLYH101, Figure S2 in File S1) and inconsistent increases in sodium absorption in CF biopsies (Figure 2, Table 2, Figure S1 in File S1) could be limitations to use of ICM as a CFTR biomarker in modulator trials. Despite these limitations, our multi-center results demonstrate good agreement in ICM parameters with single center studies that have included larger numbers of both adult and pediatric CF patients and support the hypothesis that multi-center ICM may be a feasible endpoint for future clinical trials.

In summary, our results indicate that SOPs for ICM performance can be developed across three centers, that multi-center ICM can discriminate between CF patients with severe CFTR mutations and non-CF subjects, and that ICM (performed with common SOPs) can detect partial CFTR function in CF patients. The power of multi-center ICM to detect low levels of CFTR activity compares favorably with that of multi-center NPD. CFTR activity was detectable several hours post-biopsy with moderate correlation between study sites, suggesting that the electrophysiologic aspects of ICM can potentially be centralized in the future. The assay expands upon available CFTR biomarkers by isolating CFTR currents, providing flexibility in reagents, and offering numerous CFTR-specific features that discriminate between CF and non-CF subjects. Together, these data demonstrate that ICM is a feasible biomarker for CFTR and that further development is warranted for application to clinical trials.

## Supporting Information

File S1 **Contains Figures S1-S7.** Figure S1, Examples of ICM tracings for (A) non-CF and (B) CF patients. Figure S2, Representative examples of CFTR inhibitor effects on CFTR currents in rectal biopsies. Figure S3, Effects of indomethacin on ICM parameters. Figure S4, Comparison of ICM parameters in Ringer’s buffer and RPMI + 25 mM HCO3 buffer. Figure S5, Comparison of sequential vs simultaneous agonist addition on CFTR detection by ICM. Figure S6, Comparison of four cold storage conditions on rectal biopsy performance. Figure S7, Comparison of CFTR-dependent ICM responses from forceps and suction biopsies in non-CF subjects.
(DOC)
