# Supplementary material for: Multicenter Intestinal Current Measurements in Rectal Biopsies from CF and Non-CF Subjects to Monitor CFTR Function
Source: PLoS One. 2013 Sep 10;8(9):e73905. doi: 10.1371/journal.pone.0073905 (PMC3769519; doi:10.1371/journal.pone.0073905)
Supplement: File S1 — Contains Figures S1-S7. Figure S1, Examples of ICM tracings for (A) non-CF and (B) CF patients. Figure S2, Representative examples of CFTR inhibitor effects on CFTR currents in rectal biopsies. Figure S3, Effects of indomethacin on ICM parameters. Figure S4, Comparison of ICM parameters in Ringer’s buffer and RPMI + 25 mM HCO3 buffer. Figure S5, Comparison of sequential vs simultaneous agonist addition on CFTR detection by ICM. Figure S6, Comparison of four cold storage conditions on rectal biopsy performance. Figure S7, Comparison of CFTR-dependent ICM responses from forceps and suction biopsies in non-CF subjects. (DOC) [file pone.0073905.s001.doc]

**FILE S1 – SUPPORTING INFORMATION – Clancy ICM001-002**

**Multicenter Intestinal Current Measurements in Rectal Biopsies from**

**CF and Non-CF Subjects to Monitor CFTR Function**

*John P. Clancy^1^, Rhonda D. Szczesniak^1^, Melissa A. Ashlock^2^, Sarah E. Ernst^3^, Lijuan Fan^4^, Douglas B. Hornick^3^, Philip H. Karp^3^, Umer Khan^5^, James Lymp^6^, Alicia J. Ostmann^1^, Amir Rezayat^1^, Timothy D. Starner^7^, Shajan P. Sugandha^8^, Hongtao Sun^1^, Nancy Quinney^9^, Scott H. Donaldson^9^, Steven M. Rowe^8^, and Sherif E. Gabriel^10^

**Affiliations:**

^1^Department of Pediatrics, Cincinnati Children’s Hospital Medical Center and the University of Cincinnati, Cincinnati, Ohio, United States of America

^2^Formerly Cystic Fibrosis Foundation, Bethesda, MD, USA; currently aTyr Pharma Inc., San Diego, California, United States of America

^3^Department of Internal Medicine, University of Iowa, Iowa City, Iowa, United States of America

^4^Formerly Department of Medicine, University of Alabama at Birmingham, Birmingham, Alabama, United States of America; currently Department of Medicine, Georgetown University, Washington, District of Columbia, United States of America

^5^ Department of Biostatistics, Seattle Children’s Hospital, Seattle, Washington, United States of America

^6^Genentech, Inc., San Francisco, California, United States of America

^7^Department of Pediatrics, University of Iowa, Iowa City, Iowa, United States of America

^8^Department of Medicine, University of Alabama at Birmingham, Birmingham, Alabama, United States of America

^9^Department of Medicine, University of North Carolina, Chapel Hill, North Carolina, United States of America

^10^N30 Pharmaceuticals, Aurora, Colorado, United States of America

***Corresponding Author:** J.P. Clancy, MD, Cincinnati Children’s Hospital Medical Center, MLC 2021, 3333 Burnet Avenue, Cincinnati, OH, USA 45229 Phone: (513) 636-6771 Fax: (513) 803-2813 Email: john.clancy@cchmc.org

**Results**

*Development of SOPs for ICM performance*

See manuscript text.

**Figure S1. Examples of ICM tracings for (A) non-CF and (B) CF patients.** Each line represents a biopsy, and all data in each graph are from one subject. Please see the text for details of the reagent concentrations used.

**Figure S2. Representative examples of CFTR inhibitor effects on CFTR currents in rectal biopsies** (CFTR_inh_172 – 50 μM, GLYH101 – 100 μM). Neither CFTR-specific blocker inhibited currents in a reproducible fashion. Bumetanide (Bum, 100 µM) was added to the basolateral compartment. Both blockers were able to reduce CFTR-dependent currents following prolonged incubation (data not shown).

**Figure S3. Effects of indomethacin on ICM parameters.** 10 µM indomethacin (Indo) or vehicle was added to the bath solution for twenty minutes prior to subsequent reagents. The addition of Indo significantly enhanced the changes in current in response to amiloride, forskolin (Fsk)/IBMX, and carbachol (CCh), Fsk/IBMX + CCh (biopsies from six non-CF subjects, *P* < 0.01).

**Figure S4. Comparison of ICM parameters in Ringer’s buffer and RPMI + 25 mM HCO3 buffer.** Tissues were mounted and studied in either Ringers Buffer or RPMI buffer + 25 mM HCO_3_ and placed in indomethacin and amiloride as described. No differences were identified in response to forskolin/IBMX (cAMP), carbachol (CCh), and cAMP + CCh across the two buffers (biopsies from six non-CF subjects).

**Figure S5. Comparison of sequential vs simultaneous agonist addition on CFTR detection by ICM.** Tissues were mounted and studied in RPMI buffer + 25 mM HCO_3_, and placed in indomethacin and amiloride as described. The total change in I_sc_ following the simultaneous addition of forskolin (10 µM)/IBMX (100 μM) (cAMP) + carbachol (CCh, 100 µM) was compared with the total I_sc_ change when the reagents were added sequentially (10 min between reagents). Sequential addition produced higher total CFTR currents compared with simultaneous stimulation with all three agonists (*P* = 0.01, biopsies from six non-CF subjects).

*Effects of cold tissue storage on ICM parameters*

See manuscript text.

**Figure S6. Comparison of four cold storage conditions on rectal biopsy performance.** Biopsies from five non-CF subjects (per condition) were studied by ICM immediately post-biopsy or after 18 hours in one of four buffer conditions [RPMI, perfadex (Xvivo perfusion, Goteburg, Sweden), aqix (Aqix, London, UK), and revive (Revive Organtech, Irvine, CA)] + supplemental antibiotics (penicillin 100 µg/ml, gentamicin 100 µg/ml, ciprofloxacin 20 µg/ml). ICM responses to **(A)** forskolin/IBMX (cAMP), **(B)** carbachol (CCh), or **(C)** cAMP + CCh are shown. **P* < 0.001 compared with day 01 RPMI, ***P* < 0.05 compared with day 01 RPMI.

**DISCUSSION**

**Figure S7. Comparison of CFTR-dependent ICM responses from forceps and suction biopsies in non-CF subjects. (A)** Forskolin/IBMX (cAMP) responses of forceps and suction-based rectal biopsies from three non-CF subjects, **(B)** carbachol (CCh) responses of forceps and suction-based rectal biopsies from three non-CF subjects, and **(C)** cAMP + CCh responses of forceps and suction-based rectal biopsies from three non-CF subjects. Individual forceps biopsies (blue - left) and suction biopsies (green - right) from three individuals are shown (square, circle, triangles). Mean biopsies per subject (red) are as shown, with a line connecting the values. Differences between cumulative biopsy results were not statistically significant.

**Figure S1**

**
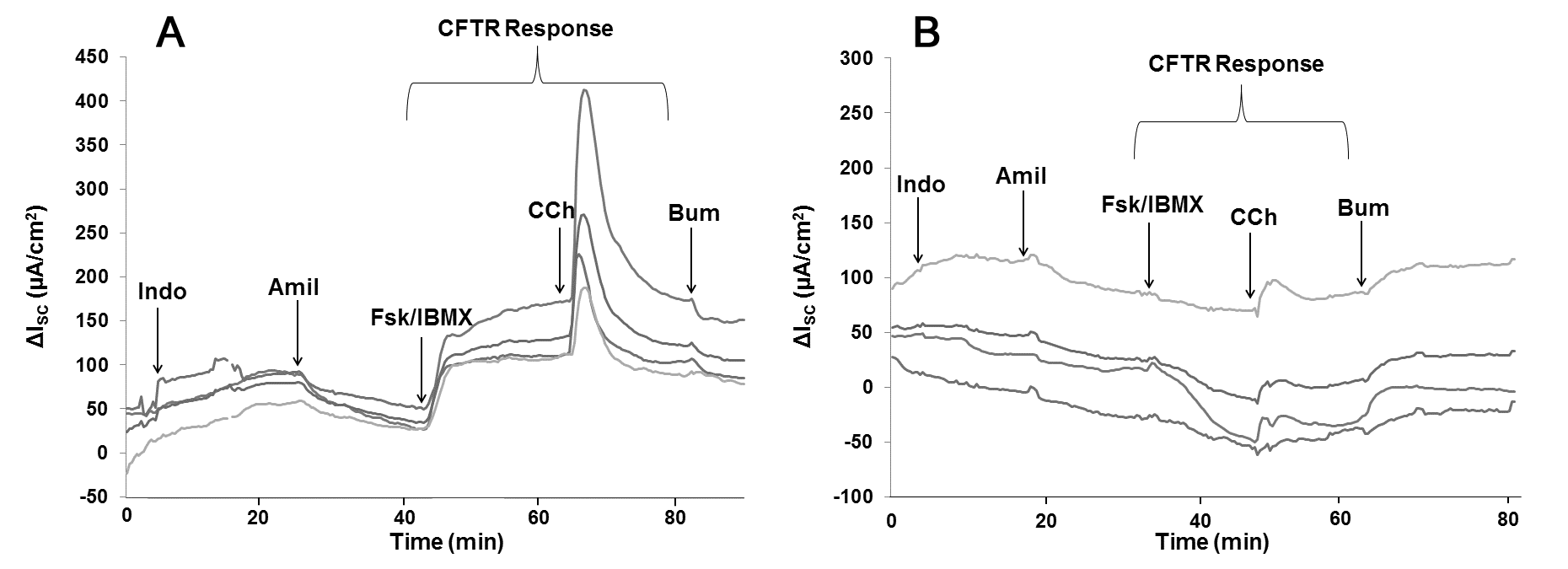
**

**Figure S2**

**
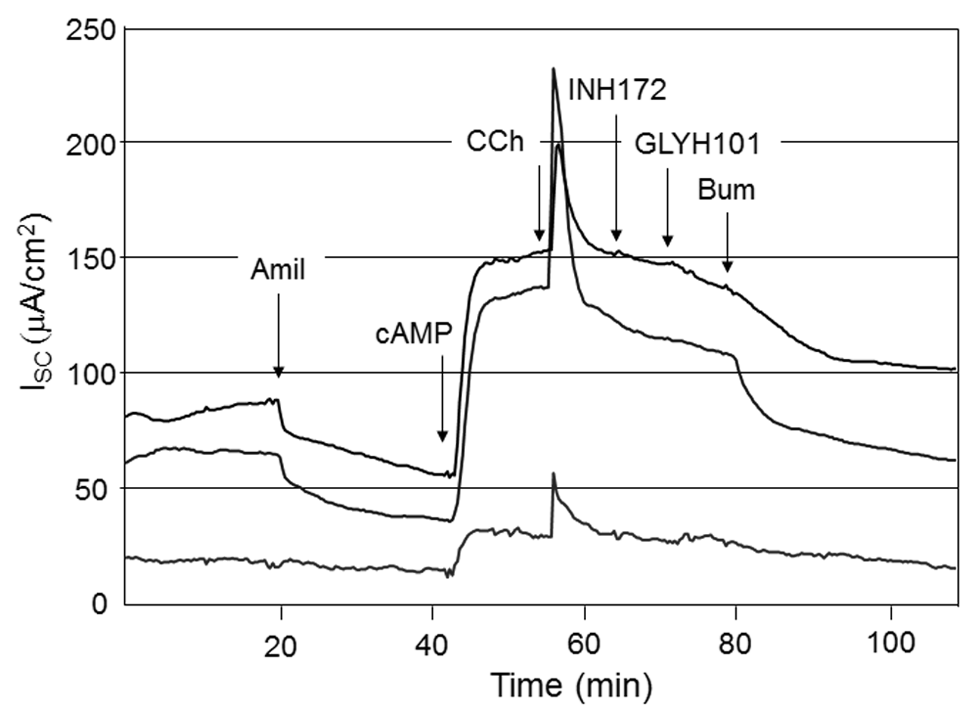
**

**Figure S3**

**
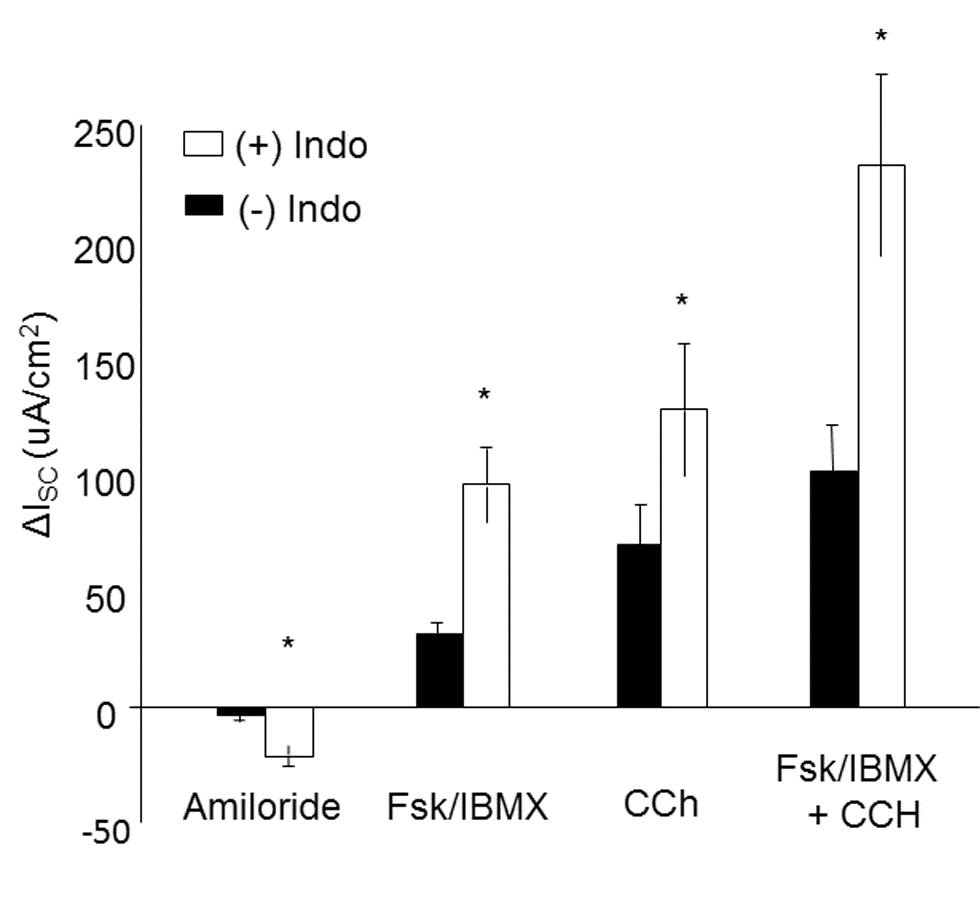
**

**Figure S4**

**
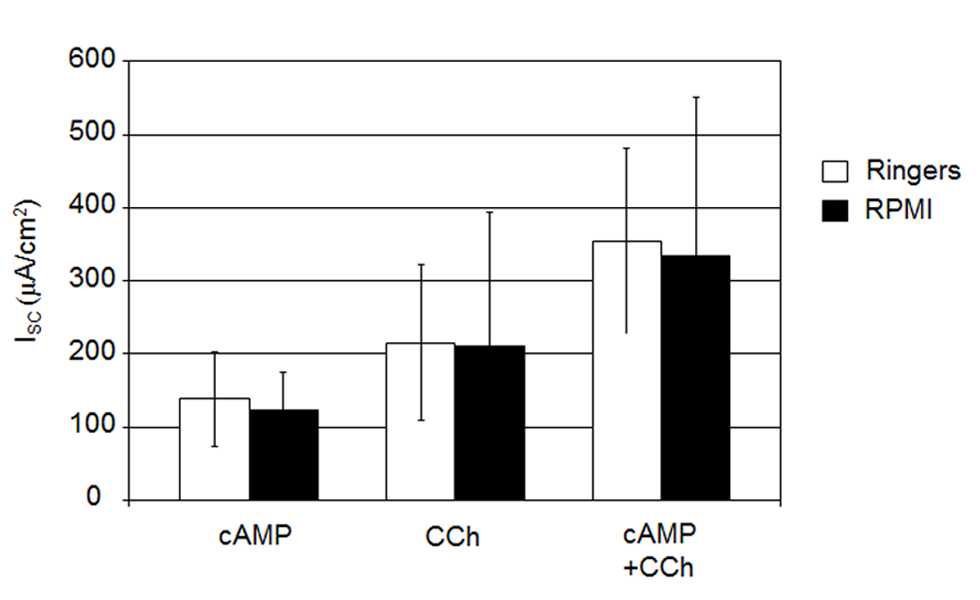
**

**Figure S5**

**
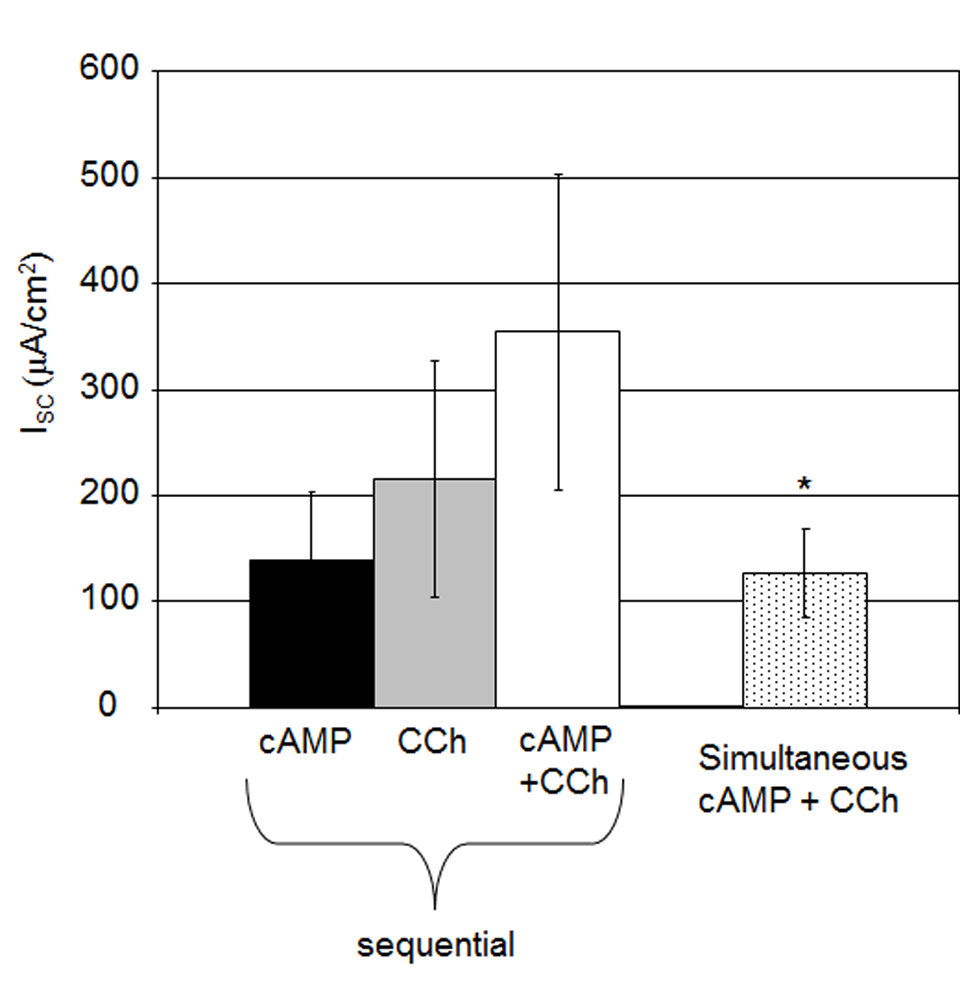
**

**Figure S6**

**
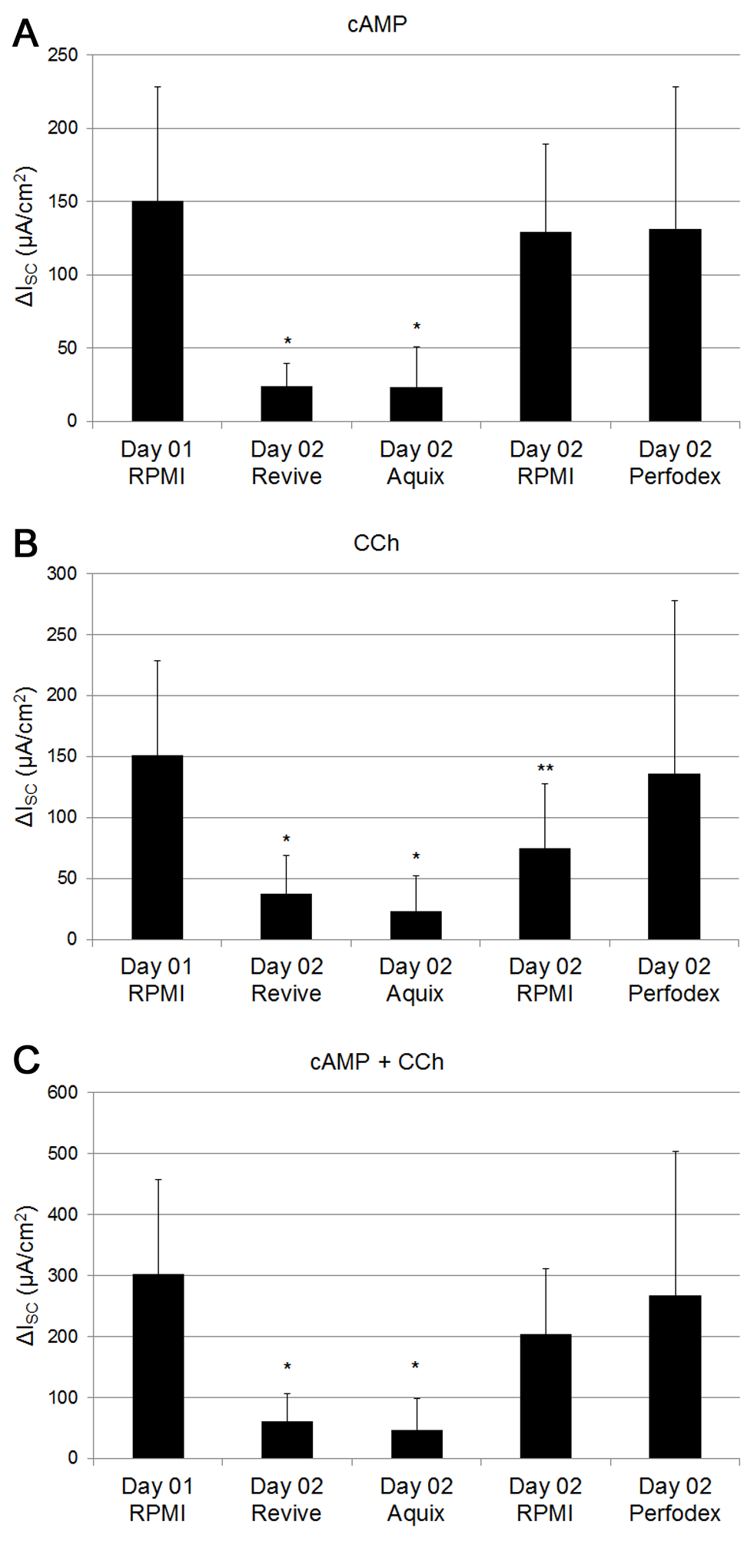
**

**Figure S7**

**
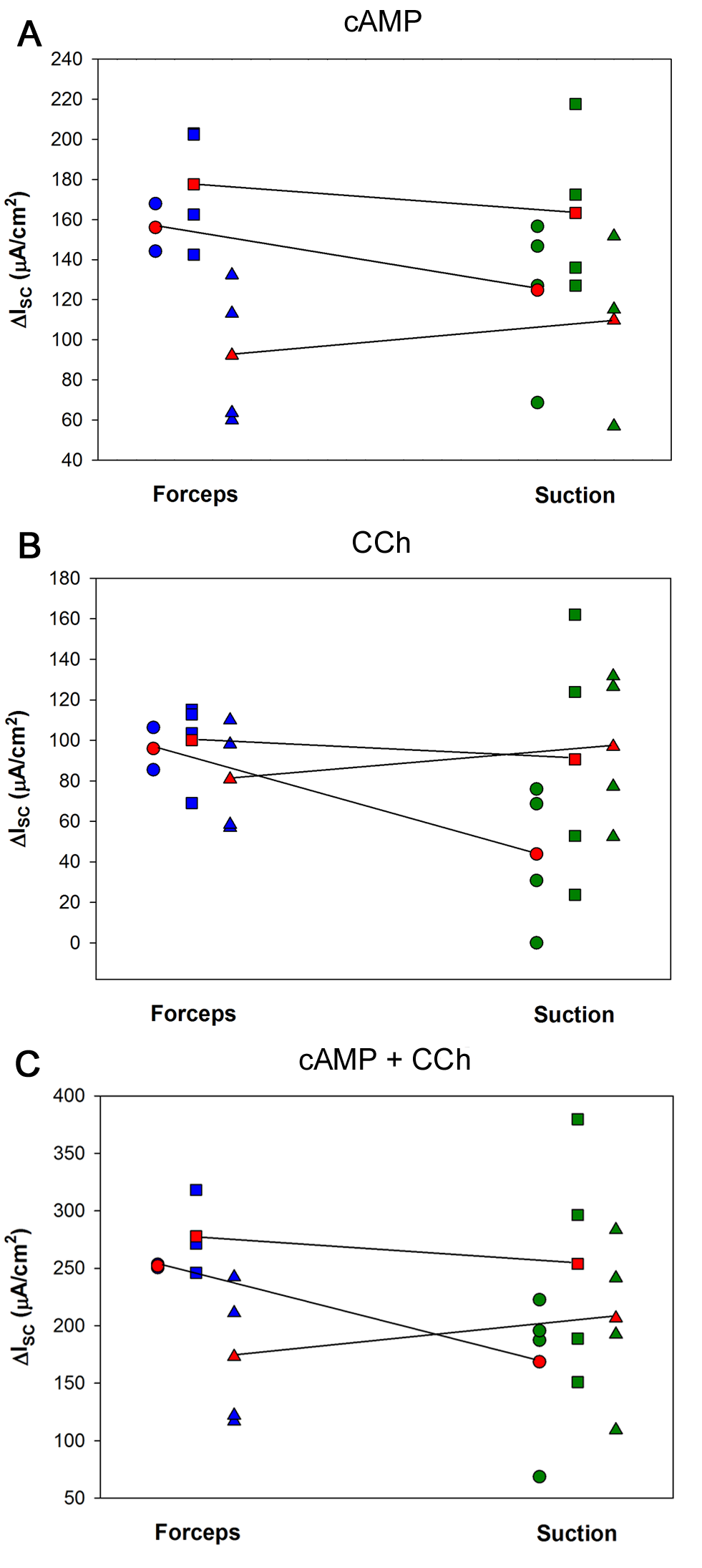
**
